# The Role of Ion Exchange Membranes in Membrane Capacitive Deionisation

**DOI:** 10.3390/membranes7030054

**Published:** 2017-09-14

**Authors:** Armineh Hassanvand, Kajia Wei, Sahar Talebi, George Q. Chen, Sandra E. Kentish

**Affiliations:** 1 Department of Chemical Engineering, University of Melbourne, Parkville, VIC 3010, Australia; ahassanvand@student.unimelb.edu.au (A.H.); stalebi@student.unimelb.edu.au (S.T.); gechen@unimelb.edu.au (G.Q.C.); 2 Key Laboratory of Jiangsu Province for Chemical Pollution Control and Resources Reuse, School of Environment and Biological Engineering, Nanjing University of Science and Technology, Nanjing 210094, China; wkjzerone@hotmail.com; 3 The ARC Dairy Innovation Hub, Department of Chemical Engineering, University of Melbourne, Parkville, VIC 3010, Australia

**Keywords:** cation exchange membrane, anion exchange membrane, desalination, carbon

## Abstract

Ion-exchange membranes (IEMs) are unique in combining the electrochemical properties of ion exchange resins and the permeability of a membrane. They are being used widely to treat industrial effluents, and in seawater and brackish water desalination. Membrane Capacitive Deionisation (MCDI) is an emerging, energy efficient technology for brackish water desalination in which these ion-exchange membranes act as selective gates allowing the transport of counter-ions toward carbon electrodes. This article provides a summary of recent developments in the preparation, characterization, and performance of ion exchange membranes in the MCDI field. In some parts of this review, the most relevant literature in the area of electrodialysis (ED) is also discussed to better elucidate the role of the ion exchange membranes. We conclude that more work is required to better define the desalination performance of the proposed novel materials and cell designs for MCDI in treating a wide range of feed waters. The extent of fouling, the development of cleaning strategies, and further techno-economic studies, will add value to this emerging technique.

## 1. Introduction

An ion-exchange membrane consists of a polymer matrix in which ionic groups are fixed to the polymeric backbone. Depending upon the charge of the ionic group, IEMs are categorized as either anion-exchange membranes (AEMs) or cation-exchange membranes (CEMs). The most common positively charged groups fixed in the former are –NH_3_^+^, –NRH_2_^+^, –NR_2_H^+^, and NR_3_^+^, which allow the transport of anions and reject cations. On the other hand, CEMs contain –SO_3_^−^, –COO^−^, PO_3_^2−^, and PO_3_H^−^, which allow the transport of cations and reject anions [1,2]. The combination of a cation and anion exchange membrane produces a third type of membrane known as a bipolar ion exchange membrane [3].

IEMs are used for mass separation, chemical synthesis, energy conversion, and storage processes [4]. The most commonly known applications are chloroalkali electrolysis and fuel cells [3,5,6], but these applications are not the focus of this paper. Rather, this paper focuses on separation processes where cations and/or anions are selectively transferred through the membrane upon applying an external electrical current [4]. This is the basis for electrodialysis (ED), Donnan dialysis, electrodialysis with bipolar membranes (EDBM), reverse electrodialysis (RED), and Membrane Capacitive Deionisation (MCDI).

**Figure 1:**
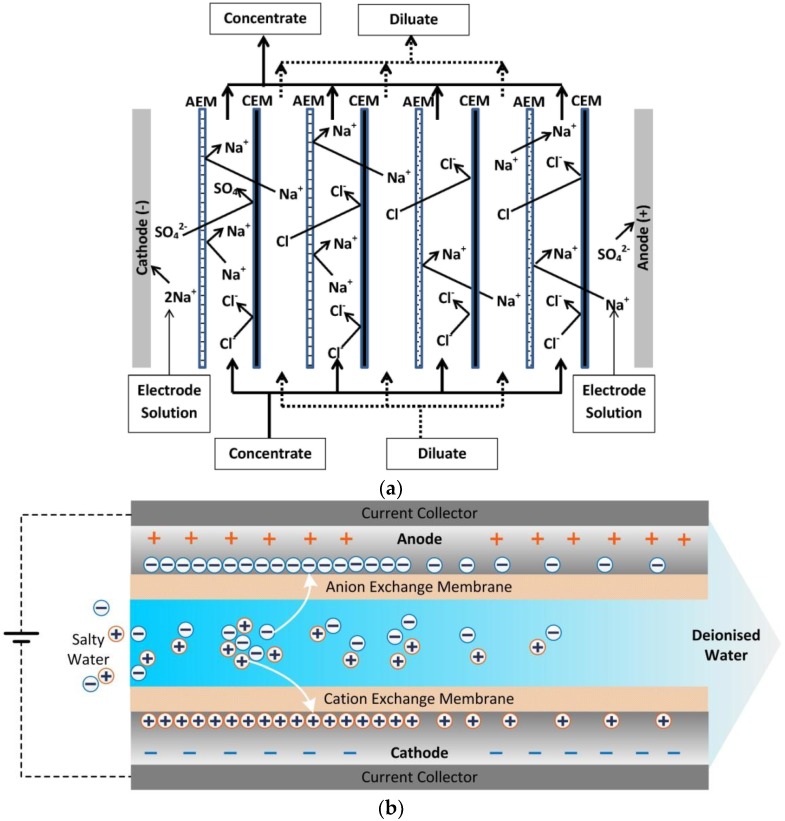
(**a**) Electrodialysis (adapted from [9]); and (**b**) Membrane Capacitive Deionisation (MCDI) during adsorption.

As shown in Figure 1a, in electrodialysis (ED), the application of an electric field causes the cations and anions present in the feed stream to migrate toward the cathode and anode, respectively [7]. The cations pass through the CEM, but are then retained in the permeate channel due to the presence of an AEM. Similarly, anions are captured between an AEM and CEM. Hence, by placing the IEMs alternatively, the feed compartments (diluate side) become depleted of ions and the permeate compartments (concentrate side) become more concentrated [8].

**Figure 2:**
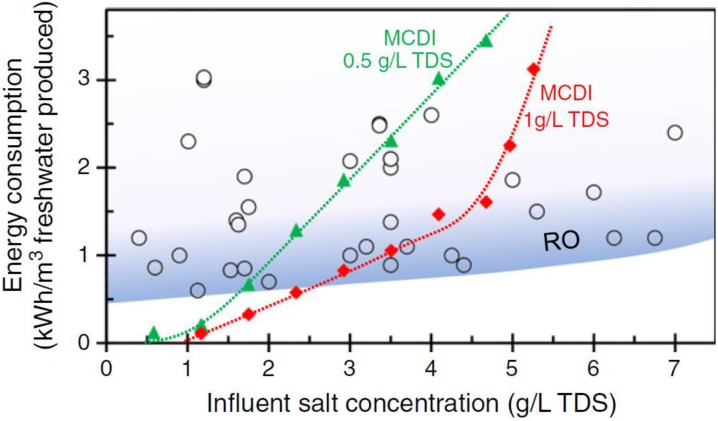
Comparison of energy consumption between MCDI and reverse osmosis. Triangles: energy consumption of MCDI to bring salt concentration to the level of 0.5 g L^−1^ Total Dissolved Solids (TDS). Diamonds: energy consumption of MCDI to reduce salt concentration to 1 g L^−1^ TDS. Circles: energy consumption of Reverse Osmosis (RO) collected from literature studies. Reproduced with permission from [16], copyright Elsvier, 2013.

While ED is considered a mature technology in water desalination, Capacitive Deionisation (CDI) is an emerging technology. This approach has very low energy consumption at low salinity, is easy to operate and is low maintenance [10,11]. In CDI, an electric field is applied to carbon electrodes, causing charged species to be adsorbed within the carbon micropores [12]. During regeneration, the electric field is removed and the adsorbed ions are released back into a brine stream. One of the drawbacks limiting the charge efficiency in CDI is co-ion adsorption, i.e., the adsorption of ions to an electrode carrying the same surface charge [13]. In 2006, Lee et al. [14] suggested introducing ion-exchange membranes in front of the carbon electrodes to reduce this effect. As depicted in Figure 1b, in a MCDI cell, an anion-exchange membrane is placed in front of the anode to block the passage of cations and a cation-exchange membrane is placed in front of the cathode to reject the anions [15]. This approach significantly limits the co-ion adsorption. It also allows for the electrical charge to be reversed during the regeneration cycle, rather than being simply turned off. Zhao et al. [16] found that MCDI can be more energy efficient than Reverse Osmosis (RO) technology, when the feed water salinity is <2 g L^−1^ Total Dissolved Solids (TDS) and effluent water TDS >0.5 g L^−1^ (see Figure 2).

This review provides a summary of recent accomplishments in the fast evolving area of MCDI and the novel IEMs being prepared for this purpose. Additionally, the effect of fouling and scaling in these systems is discussed. Recent progress in the area of ion transport through IEMs is also discussed briefly. In the final section, we describe the most recent MCDI stack designs where novel features are introduced to the conventional MCDI cell.

## 2. Ion Exchange Membranes for Membrane Capacitive Deionisation (MCDI) Applications

### 2.1. Homogeneous Ion Exchange Membranes

Within a homogeneous ion exchange membrane, the ionic groups are chemically bound to a polymeric backbone making a coherent ion-exchanger gel [1,2]. These functionalised polymers would swell greatly in water if not crosslinked; thus, crosslinking agents, most commonly divinyl benzene monomers, are also incorporated [17]. In addition to less swelling, crosslinking also results in an improved structure of the IEM that can facilitate the introduction of a greater density of fixed charge groups [18]. In a traditional production process, the divinyl monomers are mixed with a linear polymer, polymerisation initiators, and plasticisers to form a paste. This paste is then coated onto a backing fabric or net and the composite structure is heated to copolymerise the divinyl monomers. The ion exchange functionality is added after membrane formation by impregnation of chemicals such as chlorosulfonic acid, sulfuric acid, trimethylamine, or methyl iodide. However, the dangerous nature of these chemicals is resulting in an increasing tendency to instead use monomers that are already functionalised [2]. In this case, a water soluble crosslinking agent, typically formaldehyde, is used in place of divinyl benzene [19]. An alternate CEM can be prepared from a perfluorinated sulfonic acid polymer such as Nafion™ (DuPont, DE, USA).

IEMs that are commercially available for MCDI applications include Fumasep IEMs produced by FuMA-Tech GmbH (Bietigheim-Bissingen, Baden-Württemberg, Germany), Selemion IEMs produced by Asahi Glass Co., Ltd. (Tokyo, Japan), and Neosepta IEMs produced by ASTOM Co. (Tokyo, Japan) (Table 1 and Table 2). These membranes benefit from high permselectivity, low electrical resistance, and high chemical and mechanical stability [3].

Several workers have developed IEMs specifically for MCDI. In this application, the membranes can be thinner than in ED applications, as they do not need to be self-supporting. This results in lower electrical resistance. Kwak et al. [20] developed a cation exchange membrane through heat crosslinking and esterification of three monomers, 4-styrenesulfonic acid sodium salt hydrate (NaSS), methacrylic acid (MAA), and methyl methacrylate (MMA). The synthesis of a poly(vinylidene fluoride)-sodium 4-vinylbenzene sulfonate copolymer (PVDF-g-PSVBS) CEM for deionisation applications was also reported by Kang et al. [21]. Jeong et al. [22] synthesized an aminated poly(vinylidene fluride-g-4-vinylbenzyl chloride) (PVDF-g-VBC) anion exchange membrane, while Qiu et al. [23] used γ-irradiation to crosslink a thin sulfonated polystyrene film as a CEM.

### 2.2. Composite Electrodes for MCDI

The carbon electrodes used in CDI applications are generally prepared from activated carbon, or other forms of carbon such as graphene or carbon nanotubes. These carbon particles are bound together through the addition of a weakly hydrophilic polymer binder such as polyvinylidene fluoride (PVDF). Lee et al. [24] were the first to combine ion-exchange resin directly into a CDI electrode to improve the hydrophilicity of these structures. They achieved salt removal efficiencies 35% higher than a conductive carbon black/carbon composite electrode when Purolite anion exchange resin was added to both electrodes. Hou et al. [25] similarly used polyvinyl alcohol as a more hydrophilic binder.

However, later work has focused on incorporating ion exchange resin into the carbon electrode or coating it with an ion selective coating. This provides a composite structure within which the ion selectivity is comparable to separate IEM and electrode layers. Application of such electrodes can potentially result in a system that is lower in capital cost and electrical resistance than a conventional MCDI unit, but with the same current efficiency. This approach is referred to as modified MCDI (m-MCDI) [26].

**Figure 3:**
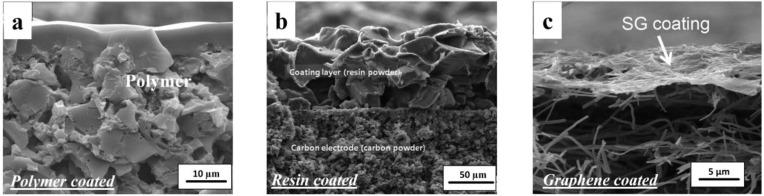
SEM images of three typical composite electrodes. (**a**) Carbon electrode coated with PVA/SSA polymer solution, copyright Elsvier, 2010; (**b**) composite carbon electrode coated with resin powder, copyright Elsvier, 2012; (**c**) Sulfonated graphite (SG) carbon nanofibre composite. Reproduced with permission from [27,28,35], copyright John Wiley and Sons, 2015.

Kim and Choi [27,29] were the first to try this approach and coated a mixture of poly(vinyl alcohol) (PVA) and sulfosuccinic acid (SSA) directly onto a carbon electrode to form a cation selective electrode (Figure 3a). The other electrode was left uncoated. This approach gave a 15 to 30% increase in specific capacitance for the electrode, while giving less electrical resistance than commercial IEMs. The same group [35,36] later fabricated nitrate-selective composite electrodes (Figure 3b) by coating anion exchange resin powder onto a carbon electrode. In MCDI experiments, they coupled this novel electrode with a standard Neosepta CMX membrane attached to the other electrode. Tian et al. [37] synthesised an anion exchange membrane electrode that combined a cross-linked PVA layer functionalised with quaternary amine groups above an activated carbon layer, while Gu et al. [38] used a similar coating on a graphene sponge electrode (Figure 3c). Qian et al. [28] used sulfonated graphene to prepare an ultrathin cation-selective coating achieving enhanced electrochemical specific capacitance and charge efficiency.

While these researchers developed only one ion selective electrode in isolation, other researchers have developed electrode pairs, generally by sulfonation and amination of a base polymer, which is then sprayed or coated onto the carbon electrode [30,31]. Liu et al. [32] similarly directly sulfonated and aminated 3D graphene. Kim et al. [33] blended and crosslinked PVA with sulfur succinic acid (SSA) and poly(styrene sulfonic acid-co-maleic acid) (PSSA-MA) to provide a CEM and used animated polysulfone for the AEM. Liu et al. [26] introduced the anion exchange polymer dimethyldiallyl ammonium chloride and cation exchange polymer polyethyleneimine (PEI) as a binder in carbon nanotube electrodes to form such a pair.

These composite electrodes appear a promising approach to reducing electrical resistance. However, more work is required to determine if they are indeed lower in cost than the traditional arrangement and to evaluate their long term performance in desalination of a wide range of charged species.

## 3. MCDI Performance Parameters

**Figure 4:**
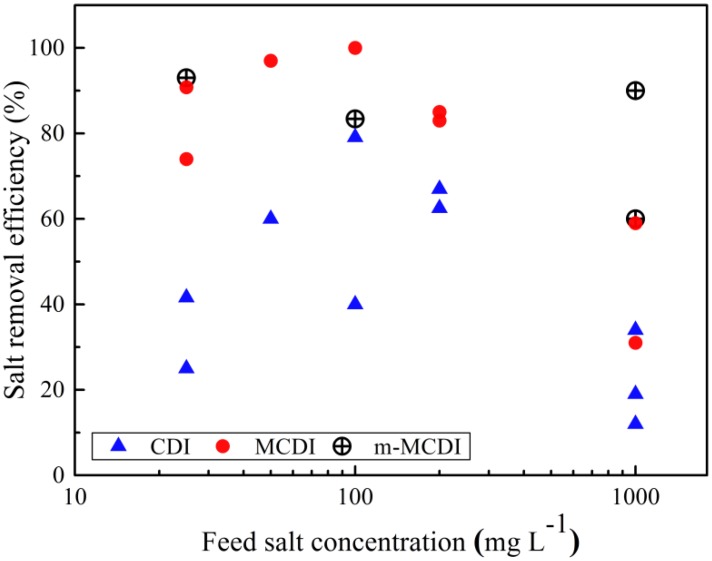
Salt removal efficiencies as a function of feed salt concentration from recent research reports on Capacitive Deionisation (CDI), MCDI, and modified MCDI (m-MCDI) [14,24,26,29,30,31,39,40,41,42].

There are several key parameters that indicate MCDI or CDI performance. The salt removal efficiency is the percentage of salt removed from the feed stream in each adsorption cycle and reflects the cell geometry, feed flowrates, charge applied, and carbon properties. In general, MCDI provides better salt removal efficiency compared with CDI due to the almost complete elimination of co-ion adsorption (Figure 4). Such efficiencies are mainly above 70% for MCDI and m-MCDI, while for CDI cells, these are mostly below 70%.

The adsorption capacity is the amount of salt adsorbed per gram of capacitive materials within the adsorption cycle. This capacity depends most strongly upon the type of electrode used, but is generally higher in direct experimental comparisons for m-MCDI or MCDI versus CDI (Table 3).

CDI and MCDI cells are operated either at constant current (CC) where the voltage varies during the cycle, or constant voltage (CV) where the current is varied. The charge efficiency (Λ) is used to characterise the CV mode and is defined as the ratio of adsorbed salt (Γ, mol g^−1^) per cycle to the charge (Σ, C g^−1^) transferred in this cycle [47]:

(1) $$\Lambda =\frac{\Gamma \times F}{\Sigma }$$

where F represents Faraday constant (96,485 C mol^−1^), Γ is the adsorption capacity, and Σ is calculated by integrating the corresponding current [48].

The current efficiency, λ, describes the CC mode and is similarly defined as the amount of ions adsorbed over current applied.

(2) $$\lambda =\frac{\left(C_{in}-C_{out}\right)VF}{It_{ads}}$$

where $C_{in}$ and $C_{out}$ are the salt concentrations (mol L^−1^) of influent and effluent, I is the applied electrical current, $t_{ads}$ is the adsorption duration, and V is the solution volume (L) [49].

**Figure 5:**
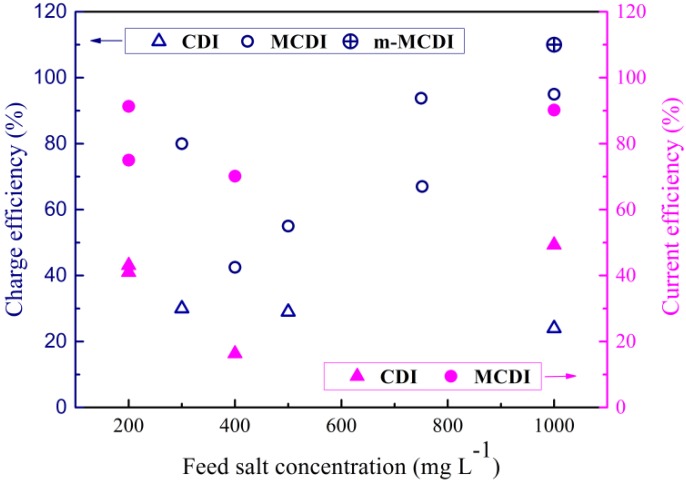
Charge and current efficiencies as a function of feed salt concentration for a range of recent studies [25,28,29,40,42,43,44,45,52,53,54].

The charge efficiencies and current efficiencies for MCDI are mostly above 50%, while those for CDI are lower (see Figure 5). The use of composite electrodes (m-MCDI) shows even higher charge efficiencies. Choi [50] found that the charge efficiency in CV mode was greater than the comparable current efficiency in CC mode, although this trend is less evident when a range of data is considered such as in Figure 5 [51]. 

The energy consumption in both CDI and MCDI is a direct function of the feed salt concentration. In a pilot scale CDI study, Mossad and Zou [55] proposed a minimum energy consumption of 1.85 kWh m^−3^ (10 kJ g^−1^) for CDI at the highest flow rate. In lab-scale tests, MCDI has been shown to provide lower energy consumption, below 1 kWh m^−3^ or 6 kJ g^−1^ [44,49]. Dlugolecki et al. [56] were able to achieve a value of 0.26 kWh m^−3^ by recovering the energy during the discharge part of the MCDI cycle. Choi [50] found that the MCDI energy consumption was much lower while operated under CC mode against CV mode. 

It should be noted that most work to date has considered only the separation of NaCl using MCDI. Choi et al. [57] considered the selective removal application of nitrate, while Ryu et al. [58] suggested a novel recovery system for lithium with a modified MCDI cell. Yoon et al. [59] proposed the use of calcium alginate as a cation exchange coating material on a negative electrode and used this approach effectively for calcium removal. In this case, the salt sorption capacity and charge efficiency were 15.6 mg g^−1^ and 95%, respectively; much higher than that for CDI (9.8 mg g^−1^ and 55%).

In another study, Choi et al. [60] used a commercial monovalent cation permselective membrane to produce calcium-rich solutions from an MCDI process, by selectively removing sodium. They mixed NaCl and CaCl_2_ at similar mass ratios and achieved a selectivity of 1.8 (removal of Na^+^ to that of Ca^2+^). This selectivity fell at higher feed TDS concentrations, lower pH values, lower applied voltage, and longer operation time. The authors compared this approach to the use of nanofiltration (NF) and argued that the NF approach was unable to produce a divalent-rich solution. However, this result is not consistent with other literature sources that do indicate NF can provide divalent selectivity [61,62,63,64]. The authors also compared the electrical energy consumption in MCDI to that of NF. With specific energy consumption of around 0.2 KWh m^−3^, MCDI was comparable with NF only at low salinity and water recoveries. However, it is worth mentioning that Choi et al. [60] considered the electrical energy consumption during both during adsorption and desorption. The use of energy recovery systems during MCDI regeneration can reduce this energy requirement [56]. Overall, there is limited data on MCDI with such alternative salts, making this a fruitful area for further research.

The research outcomes discussed in this section are also mostly collected from lab scale MCDI units. More work is required to test these membranes at a larger scale. van Limpt et al. [65] are one of the few groups to report results from commercial MCDI systems. They monitored the operation of two MCDI modules in series (each providing 6 m^2^ cell area) on cooling water recycle streams. The modules were operated under constant current conditions for more than six months at two different site locations. MCDI operation resulted in 70% conductivity removal, 83% water recovery, and savings of 85% in chemical usage. High water recovery was achieved by lowering the flow rate during regeneration. An energy consumption of 0.1 to 0.2 kWh m^−3^ was reported, which is significantly lower than that of RO in brackish water deioniziation (0.85–1.55 kWh m^−3^). 

## 4. Fouling

Fouling is an unfavorable phenomenon caused by the attachment of a substance or a living organism to the surface of the membrane. Mikhaylin et al. [66] classified IEM fouling into three categories—colloidal fouling, organic fouling, and biofouling. Non-dissolved suspended solids such as aluminium silicate clays contribute to colloidal fouling, while organic fouling is caused by organic materials such as oils, proteins, and humic acid. Biofouling is initiated by deposition of bacteria or algae onto the membrane, where these microorganisms proliferate and exude extracellular polymeric substances [67,68]. Alternatively, scaling occurs when a dissolved species precipitates onto the membrane surface.

### 4.1. Fouling in Capacitive Deionisation

In CDI mode, fouling directly affects the carbon electrodes. Mossad et al. [69] used the sodium salt of humic acid as a model foulant of CDI electrodes and reported a fall in salt removal efficiency and feed flow rate over 30 h of operation. They attributed this deterioration to organic fouling blocking the carbon pores. Energy consumption increased by 39% with the introduction of only 10 mg L^−1^ of organic matter to the feed composition. For a feed containing NaCl and humic acid, neither hydraulic or acid cleaning was effective in recovering the flow rate; an alkaline solution, however, could recover 86% of the initial flow rate [69]. For a feed containing both humic acid and a range of divalent salts, hydraulic cleaning was less effective, with only 62.5% of the flow rate recovered with alkaline cleaning. These researchers also observed that the amount of iron in the effluent during cleaning was significantly higher than the iron concentration in the feed, suggesting that Fe ions accumulated more readily than Ca or Mg ions on the carbon electrodes. 

Zhang et al. [70] similarly reported an increase in energy consumption during the long-term operation of two inland brackish water desalination CDI units in Australia. It was suggested that dissolved organic matter should be removed prior to the CDI unit to maintain the unit sustainability and efficiency [69]. These researchers [70] cleaned their CDI unit with 0.01 M citric acid for calcium and magnesium scaling, followed by 0.01 M NaOH solution to remove organic fouling to recover the cell performance.

Wang et al. [71] observed a deterioration in CDI desalination and regeneration performance in the presence of humic acid in domestic wastewater biotreated feed. Using impedance spectra, they calculated the resistance of fouled and virgin electrodes as 1.7 and 1.2 Ω cm^−2^, respectively. They reported that cleaning with water was capable of removing protein-like substances, while 0.01 M NaOH was most effective in the removal of humic-like substances from the carbon surface.

### 4.2. Fouling of Ion Exchange Membranes

To the best of our knowledge, Kim et al. [52] is the only research group who have studied the use of MCDI technology in the presence of foulants. They observed a decrease in salt removal efficiency in the first 6 cycles of operation when using a solution of 17 mM NaCl containing 5 and 10 mg L^−1^ of octane as the model foulant. However, this efficiency reached a stable value in the four subsequent cycles. They attributed this trend to octane reaching adsorption equilibrium on the IEM surface. They assumed octane fouling interrupted the access of ions to the carbon electrodes, lessening the salt removal efficiency. 

It is anticipated that fouling of IEMs in the MCDI arrangement should generally be less damaging to process operations than fouling of the electrodes in CDI arrangements. Further, we anticipate that fouling in these systems should replicate trends observed in electrodialysis studies, so the following section summarizes literature in this field.

In most electrodialysis studies, peptide and protein fouling is observed on the AEM [1,72,73]. This reflects the fact that these species are generally negatively charged and hence accumulate in the boundary layer near the surface of this membrane. Conversely, Langevin et al. [74] reported that CEM membranes are doubly more prone to peptide fouling in comparison to AEMs. This contrasting result probably arises from the fact that these authors completed their investigations in the absence of electrical current. Therefore, the interaction between the charged amino acids and the membrane’s fixed charged groups became the only governing factor. With systems involving proteins, the process should also be maintained at temperatures lower than 40 °C to avoid protein denaturation and agglomeration [75].

Lee et al. [76] used zeta potential to obtain the surface charges of three organic foulants, humate, bovine serum albumin (BSA), and sodium dodecylbenzenesulfonate (SDBS). They then used batch equilibrium experiments to determine the adsorption capacity of these negatively charged organic foulants on a Neosepta AMX membrane and fitted the results to Langmuir isotherms. Among the three model foulants, SDBS showed the highest adsorption capacity, even though the humate was more negatively charged. In subsequent ED trials, the SDBS (at 52 mg L^−1^) also caused a greater increase in membrane resistance and decline in current efficiency, relative to 1.0 g L^−1^ of humate and BSA. Lee et al. [76] then examined the reversibility of the fouling step by running three experiments in a row, comparing the ED performance before and after fouling without the application of any cleaning agent. They observed that the organic fouling on AMX was not reversible; it was adsorbed chemically to the membrane. 

Lindstrand et al. [77] investigated the fouling of a surface active fatty acid (octanoic acid), a surfactant (SDBS) and an alkaline bleach plant filtrate on Selemion AMV and CMV membranes. While the CEM resistance increased marginally due to scaling with the bleach plant filtrate, that of the AEM increased significantly in the presence of octanoic acid, due to electrostatic attractive forces. The authors argued that this fouling was more significant at surfactant concentrations approaching the critical micelle concentration. The AEM fouling was also more severe for octanoic acid at pH 3–3.5 than at pH 9. Since octanoic acid is not dissociated at the low pH values, they were expecting to observe similar fouling on both the AEM and CEM, but the CEM was not affected. They argued that the repulsion of the electrical dipole in the acid molecules by the CEM fixed charges outweighed the hydrophobic forces. However, it is also likely that the electric field caused the dissociation of the octanoic acid even at this lower pH, as has been observed by our own group and others for similar weak acids [78].

The pH of the feed solution promotes different types of fouling to take place. Diblíková et al. [79] reported protein precipitation on the AEM when processing cheese whey at a diluate pH of 4–5. It is known that at such pH, β-lactoglobulin has low solubility [80]. In addition, the negatively charged whey proteins cannot pass through the AEM due to their large size and thus precipitate on the surface of the membrane [81,82]. Under acidic conditions, the presence of Ca^2+^ and CO_3_^2−^ in the feed solution also promotes the precipitation of proteins on the CEM [82,83]. Several researchers reported that the gel-like protein layer noticed on AEM during whey demineralization is reversible, as it detaches easily during equipment disassembly [81,84]. However, Bleha et al. [85] argued that protein fouling becomes irreversible once it reacts with functional groups of the IEMs. 

Mineral fouling (scaling) occurs most readily on the CEM in an acidic environment and upon the AEM in a neutral or basic environment where the solubility of hydroxides and carbonates reduces [8,66,75,82,86,87]. Increases in temperature also exacerbate the precipitation of calcium salts due to their well-known reverse solubility [88]. The presence of certain ions in the feed solution can further promote the precipitation of other components. Bazinet and Araya-Farias [86] noted that when a solution containing CaCl_2_ and Na_2_CO_3_ were used, calcium hydroxide was the main deposit, while no calcium carbonate was detected. This was explained by the absence of magnesium in the solution, as magnesium is required to induce calcium carbonate nucleation.

Fouling in Electrodialysis Reversal (EDR) systems may provide a better analogue to MCDI, as in both operations the DC current is periodically reversed [89]. Vermass et al. [90] demonstrated the effectiveness of polarity reversal in EDR to remove multivalent ions and organic fouling. However, they reported that not all fouling is reversible and a combination of antifouling strategies must be employed for long-term charge efficiency stability [90].

### 4.3. Alterations to Membrane Chemistry

Salts in the feed solution can alter the counter-ion concentration within the membrane itself and thus affect performance. For example, Ayala-Bribiesca et al. [84] showed that calcium present in the feed solution could replace the counter-ion of the membrane and hence change its electrical conductivity. Shee et al. [91] similarly observed changes in the membrane composition in response to changes in the feed. Langevin et al. [74] observed that a CEM pretreated with acid (1 M HCl) was more prone to fouling in a soy protein hydrolysate solution than membrane pretreated in an alkali or neutral solution. This was attributed to the reaction of the H^+^ counter-ion within the membrane with this negatively charged species.

A number of researchers have shown that strongly alkaline solutions will damage ion exchange membranes [92,93]. Sata et al. [92] investigated the effect of NaOH concentrations ranging from 3.0 to 9.0 N on common AEMs. They showed that while all membrane showed a loss of anion exchange functionality, the *N*-methyl-pyridinium groups were more rapidly degraded, compared with benzyl trimethylammonium groups. The degradation reactions were slower at low concentrations of NaOH and lower temperatures. Membranes based on a polysulfone backbone weakened mechanically upon alkali exposure, while those made with a polyethylene fabric retained their mechanical strength. Similarly, Vega et al. [94] reported that the extent of damage to a Neosepta AMX membrane was less under low KOH concentrations (pH 10) than at higher concentrations (pH 14). This membrane is reinforced by ploy(vinyl chloride) fabric and alkali exposure that caused both the color to darken and mechanical strength to weaken due to dehydrochlorination of this fabric. Garcia-Vasuez at al. [95] showed that while AEMs exhibit both loss of functional groups and polymer backbone integrity, the sulfonate groups on the CEM are not directly affected, with only backbone chain scission occurring. 

**Figure 6:**
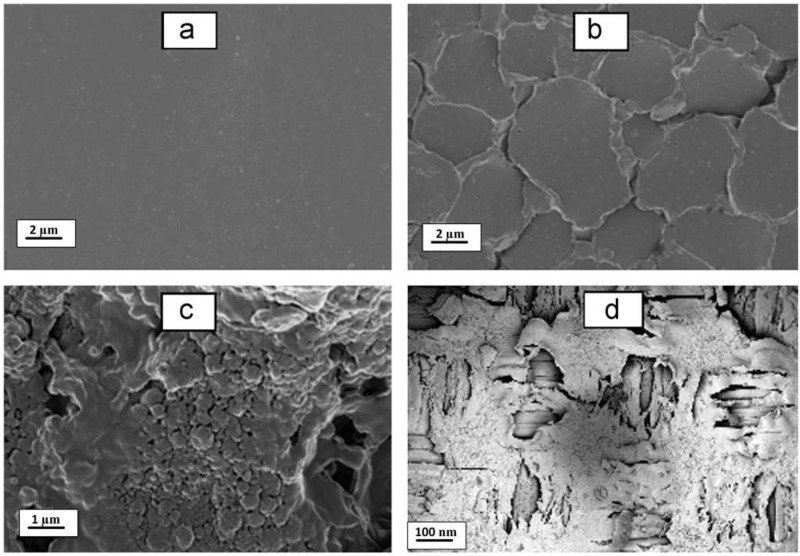
SEM micrographs of Neosepta^®^ membranes. New CEM (**a**); used CEM (**b**) and used AEM (**c**,**d**). Reproduced with permission from [96], copyright Elsvier, 2013.

Ghalloussi et al. [96] analysed the structure and physiochemical properties of four different ion exchange membranes after 2 years of ED operation with a food industry solution containing organic acids. They found that the AEMs were most severely damaged, with evidence of organic colloidal particles being sorbed into the membrane structure itself. This led to increased water content and thickness, greater surface hydrophilicity, as well as structural damage (see Figure 6). Conversely, there was evidence of a loss of sulfonic acid functional sites from the CEM, leading to a decrease in water content, greater hydrophobicity, and a more dense structure. Both membranes suffered a loss in specific conductivity and ion exchange capacity, with the CEM also experiencing a loss in permselectivity.

### 4.4. Cleaning Solutions

Physical cleaning of membranes involves forward or backwashing, air sparging, or vibration. Those processes have proven to be effective for pressure-driven membrane processes, but not for dense non-porous ion-exchange membranes such as used in MCDI processes [97]. For these operations, chemical membrane cleaning is preferred as it utilizes chemical reactions to weaken the bonds between the foulants themselves and the foulant-membrane surface [88]. There are five groups of chemicals that can be used as cleaning agents for membrane processes, namely: alkalis, acids, metal chelating agents, surfactants, and enzymes—each targeting different types of deposits. The type of cleaning chemical to be utilized is usually selected based upon the nature of the foulant, the compatibility of the chemical with the processing equipment, chemical availability, cost, and safety [88,98]. However, chemical cleaning has many disadvantages, namely: membrane damage, usage of large quantities of chemicals, and waste generation. 

Generally, alkaline cleaning agents are known for their ability to remove organic foulants, such as peptides and proteins, as the functional groups of those foulants deprotonate at pH 11 [74], while an acid clean can remove mineral deposits. Similarly, chlorinating agents are often used to remove biological fouling from membranes. However, Garcia-Vasuez at al. [95] has shown that both AEM and CEM membranes degrade upon exposure to sodium hydochlorite, losing electrical conductivity, ion exchange capacity, and mechanical strength. Furthermore, although the use of sequestering agents, such as EDTA, are found useful for mineral deposit removal from pressure driven membranes [88], they should be used with care for ion-exchange membranes, as they may tend to remove the charged ions from the membrane structure. 

Cleaning thus needs to be carried out with some care for ion exchange membranes, as they are readily degraded by extremes of pH, particularly alkalinity, as discussed in Section 4.3. The American Water Works Association claims that 2–5% HCl solution, 3–5% NaCl solution adjusted to a pH of 8–10 using NaOH, and 10–50 mg L^−1^ chlorine solution are the only chemicals that should be utilized for ion-exchange membranes in ED stacks [99]. Langevin and Bazinet [74] investigated the effect of 2% and 5% NaCl solutions as cleaning agents. They found that such solutions were able to remove larger foulant particles (>900 Da) from a Neosepta CMX membrane, but was less effective on smaller particles.

The interaction between membrane charge and foulant should be taken into account [100], as alkali and acid cleaning agents have the tendency to replace the equilibrated counter-ions in the membrane with H^+^ and OH^−^, respectively. This can cause problems in later operation as these ions are replaced with other salts. Research has shown that AEMs are more sensitive to cleaning chemicals compared to CEM. Garcia-Vasquez et al. [101] repeated an ex-situ clean-in-place (CIP) cycle for 400 h on an AEM and a CEM both having the same materials as the support and binder, but different ion-exchange groups. The cycle consisted of soaking for 30 min in 0.1 M HCl; rinsing with deionized water; soaking for 30 min in 0.1 M NaOH and rinsing again with deionized water. The cycle resulted in an increase in the hydrophilicity of the AEM causing higher water uptake, greater membrane electrical conductivity, formation of pores and cavities in the membrane, loss of toughness and flexibility, and modification of the binder material. Less damage was noted for the CEM, which was attributed to the minimal change in water uptake as the charged groups changed from Na^+^ to H^+^. 

## 5. Modelling MCDI Behaviour

**Figure 7:**
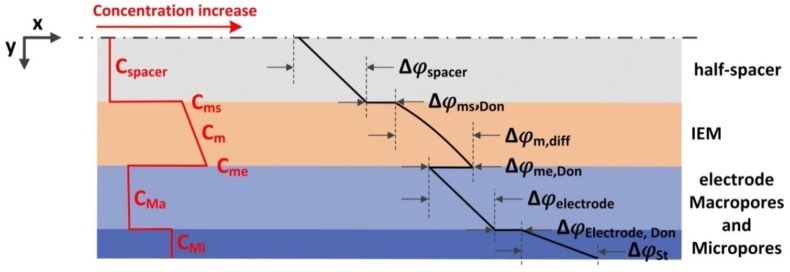
Schematic illustration of counter-ion concentration (*C*) and dimensionless voltage (φ) distribution over a half-cell within an MCDI stack. Subscripts ms, m, me, Ma, and Mi stand for membrane/spacer interface, membrane phase, membrane/electrode interface, carbon macropores, and micropores, respectively. Reproduced with permission from [103], copyright Elsvier, 2017.

An MCDI stack can be modelled by considering the resistance to flow of ions from the flow channel, through the IEMs, through the macropores in the carbon, and into the carbon micropores (Figure 7). The modified Donnan theory proposed by Biesheuvel et al. [102] can be used to describe the ion storage within these pores. This theory specifies the relationship between the concentration in the micropores with the Donnan potential drop of these micropores ($\Delta \varphi_{electrode,donnan}$) and the Stern layer potential drop ($\Delta \varphi_{St}$) within the electrical double layer (see Figure 7). However, in this Section, we focus on the role of the IEMs, as these are the defining difference between CDI and MCDI.

The flux of counter-ions $\left(J_{+,m}\right)$ through the IEM can be described by the Nernst-Planck equation. In the absence of convective flow, this equation is composed of a diffusion term driven by a chemical potential gradient, and an electromigration term driven by an electrical potential gradient:

(3) $$J_{+,m}=-D_{+,m}\left(\frac{dC_{+,m}}{dy}+C_{+,m}\frac{dln\gamma_{+,m}}{dy}\right)-D_{i,m}\frac{z_{i}C_{+,m}F}{RT}\frac{d\psi_{m,diff}}{dy}$$

where $C_{+,m}$ is the concentration, $D_{+,m}$ is the diffusion coefficient and $\gamma_{+,m}$ is the activity coefficient of the counter-ion, $C_{fix,m}$ is the concentrations of fixed charge groups in the membrane, $\psi_{m,diff}$ is the diffusion electrical potential, $z_{i}$ is the charge of the ion, *R* is the universal gas constant (8.314 J mol^−1^ K^−1^), *T* is the temperature (K), and y is the distance perpendicular to the membrane surface. 

In general, the fixed charge group concentration [104,105,106], affinity of the competing ions [107,108,109], and water uptake of the membrane [106,110] are known to affect the ultimate ion concentration within the membrane $\left(C_{i,m}\right)$. The ion activity coefficients $\left(\gamma_{+,m}\right)$ in the membrane cannot be measured directly and in most studies, these are either assumed to be unity, or equal to that in the solution (i.e., $\gamma_{+}$/$\gamma_{+,m}$ = 1) [111,112,113]. However, the activity coefficient of the counter-ions is indeed expected to be significantly lower than that of the free aqueous solution [114], due to the high concentration of the counter-ions ($C_{+,m}$) within this phase [115]. In contrast, the concentration of co-ions in the membrane ($C_{-,m}$) is much lower hence this activity coefficient is expected to be closer to unity. Recent advancements have been made by Kamcev et al. [116] to relate the ion activity coefficients to the concentration of fixed charges in the membrane ($C_{fix,m}$) and a dimensionless linear charge density (ζ) of the polymer chains, based on Manning’s counter-ion condensation theory for polyelectrolyte solutions [15]. For a 1:1 electrolyte $\gamma_{+,m}\gamma_{-m}$ is:

(4) $$\gamma_{+,m}\gamma_{-m}=\left[\frac{X/\zeta +1}{X+1}]\cdot \exp[-X/\left(X+2\zeta \right)\right]$$

where X is the ratio of fixed charge group to mobile ion concentration (i.e., X=Cfix,mC−,m).

The Meares model is widely used to describe the co-ion diffusion coefficient through the swollen polymer network ($D_{-,m}$) [114]:

(5) D−,mD−,s=(1−Φp1+Φp)2

where $D_{-,s}$ is the diffusion coefficient of the co-ion in the solution and $\Phi_{p}$ is the polymer volume fraction in the membrane [117,118,119]. For counter-ions, the electrostatic effect from the fixed charge groups cannot be neglected. To account for this effect, Manning’s model [120] can again be applied, by assuming that ion diffusion is affected by the local electric field and that condensed counter-ions have no mobility. This results in the extended Meares/Manning Model [121] for the counter-ion diffusion coefficient of 1:1 electrolytes $D_{+,m}$:

(6) D+,mD+,s=(Xζ+1X+1)(1−13A(1;Xζ))(Φw2−Φw)2

where $\Phi_{w}$ is the water volume fraction in the membrane, the parameter *A* is dependent upon measurable parameters X and ζ.

While a range of research groups have established techniques to evaluate the concentration, activity, and diffusivity coefficients in IEMs, this knowledge has not been widely employed in MCDI models. The application of more accurate membrane related parameters will further improve the ion transport models evolving for MCDI. 

## 6. MCDI Novel Stack Developments and Outlook

The search for novel MCDI configurations has led to the introduction of various technologies where researchers aimed to overcome the restrictions and drawbacks associated with conventional MCDI systems. In this section, recent developments in this area are described to frame future directions in this field. 

### 6.1. Radial Deionisation (RDI)

Atlantis Technologies are currently commercialising an MCDI cell which has been modified to provide up to 100 electrode pairs in a cylindrical arrangement [122]. This approach has been tested on phosphate mining wastewater, shale gas produced water and landfill leachate and been shown to effectively remove trace metals such as selenium, mercury, arsenic, and uranium, in addition to more common salts [123].

### 6.2. Flow Chamber Modification

**Figure 8:**
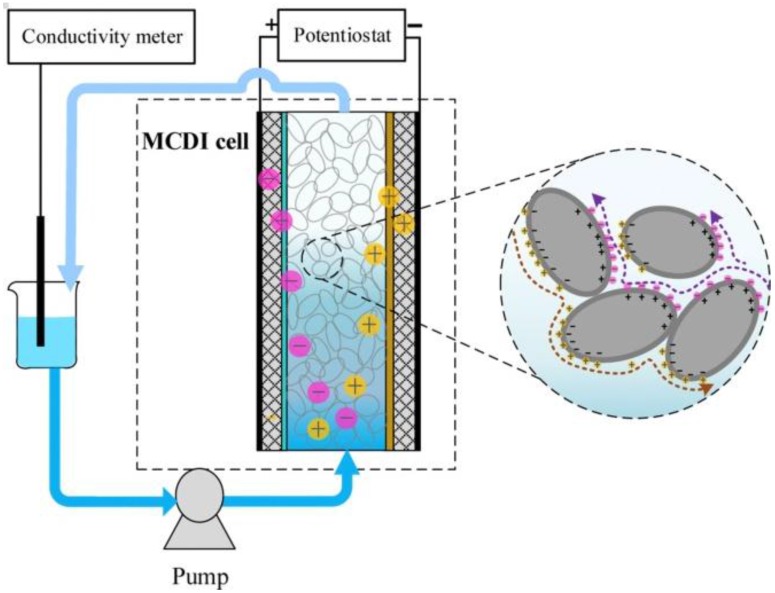
Schematic diagram of GAC (granular activated carbon)-MCDI as depicted by Bian et al. [124]. Reproduced with permission, copyright Elsvier, 2015.

In an approach similar to electrodeionisation, Liang et al. [42], filled the flow channel in an MCDI cell with ion exchange resin to reduce the electrical resistance at low salinities. This approach gave a salt removal efficiency of 90%, higher than that of either MCDI (60%) or CDI (19%) at comparable conditions. Similarly, Bian et al. [124] filled the flow chamber with granular activated carbon (GAC) (as shown in Figure 8). They observed an enhanced desalination rate compared with a conventional MCDI cell, which was attributed to a lower electrical resistance within the flow channel, as well as the introduction of additional storage sites within the GAC. 

The approach was most effective for NaCl solutions of <200 mg L^−1^. A smaller GAC particle size (0.4–0.8 mm compared with 2–5 mm) showed a better desalination rate, which was attributed to a lower energy barrier for ion transport from the bulk to the GAC pores [124]. Using non-conductive glass beads was less effective, indicating that the GAC was not just acting to increase turbulence. Conversely, while porous graphite granules showed similar ohmic resistance to the activated carbon, these could not match the desalination rate of regular MCDI due to the higher ionic resistance.

### 6.3. Flow-Electrodes Capacitive Deionisation

**Figure 9:**
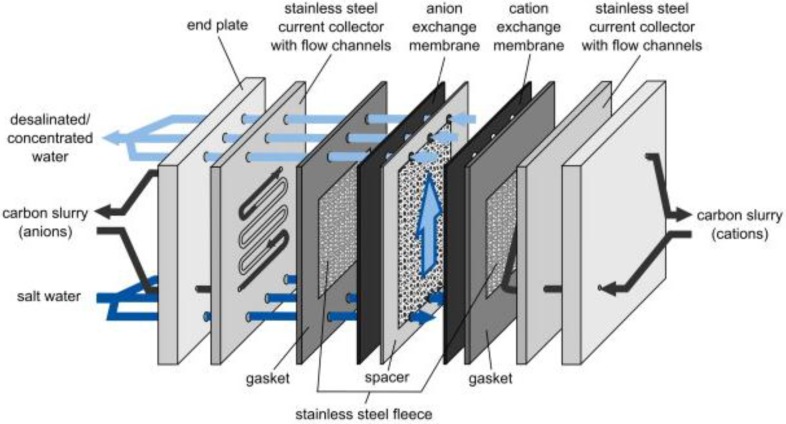
Schematic diagram of Flow-electrode capacitive deionisation (FCDI). Reproduced with permission from Gendel et al. [126], copyright Elsvier, 2014.

In 2013, Jeon et al. [125] introduced a novel configuration called flow-electrode capacitive deionisation (FCDI) in which a flowing carbon suspension replaced the solid carbon electrodes. As depicted in Figure 9, in FCDI, the ions are adsorbed into the suspended carbon materials flowing between the current collectors and ion-exchange membranes. 

This approach allows capacitive deionisation to operate in a continuous mode, rather than in adsorption and desorption cycles [125,126]. Further, whereas in a conventional MCDI process, the ion sorption capacity is restricted to the size of the fixed carbon electrodes, the ion exchange capacity of an FCDI system can be enhanced by altering the flow path size and flow rate of carbon slurry [127,128].

**Figure 10:**
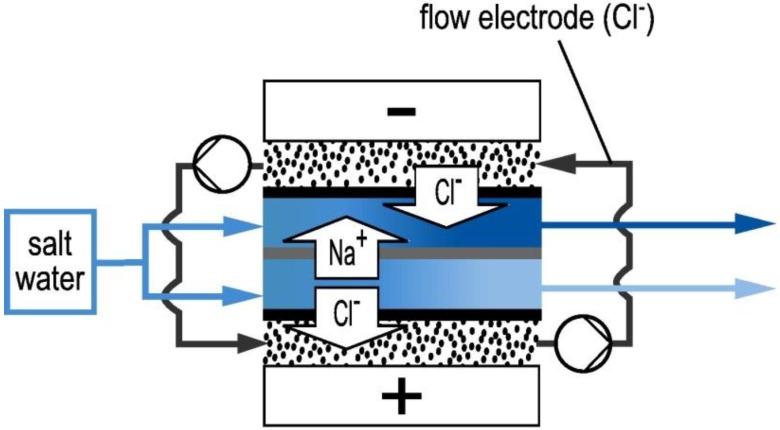
Single module flow-electrode capacitive deionisation concept as depicted by Rommerskirchen et al. [129]. Reproduced with permission, copyright Elsvier, 2015.

To regenerate the carbon suspensions, some research groups simply mix the suspension collected at the anode with that of the cathode and then let the carbon materials settle [125,127]. Alternatively, Gendel et al. [126] coupled two FCDI cells with opposing electrical potentials in which desalination and regeneration occurred separately. Later, Rommerskirchen et al. [129] developed a smart design by combing the desalination and regeneration cells into a single electrosorption module. In this case, one type of ions transfers through the ion-exchange membrane placed in the middle of the cell from a diluate compartment to a concentrate compartment. The opposite ion is adsorbed into the carbon slurry flowing on the diluate side (see Figure 10). Then, by circulating the carbon suspension into the concentrate side, the adsorbed ion is desorbed back in to the brine stream [129].

Gendel el al. [126] examined the effect of flow rate and water splitting ratio between the desalination and regeneration cells in FCDI, while Porada et al. [127] studied the effect of carbon content and water residence time. The latter reported that while salt removal rate increased with carbon content, beyond 20 wt % the flowing slurry was prone to clogging. Yang et al. [130] demonstrated that dispersing carbon particles in 2 wt % NaCl solution resulted in significantly improved desalination efficiency compared to the use of deionized water. Ma et al. [131] developed redox-active flow electrodes with the addition of aqueous hydroquinone and benzoquinone to increase the charge transfer efficiency. Further research and long-term performance data will help to determine the optimum composition of these suspensions, with high conductivity and low viscosity to achieve high charge transfer and avoid clogging. 

The authors believe that the introduction of flow-electrodes is a major step forward towards the commercialization of MCDI since it enables continuous operation and provides higher water recovery. However, Hoyt et al. [132] are among the few who have modelled the flowing carbon slurry. We believe this area requires more attention to justify the significantly higher salt adsorption and to predict the performance of FCDI under different operational conditions.

### 6.4. Hybrid Capacitive Deionisation (CDI) 

**Figure 11:**
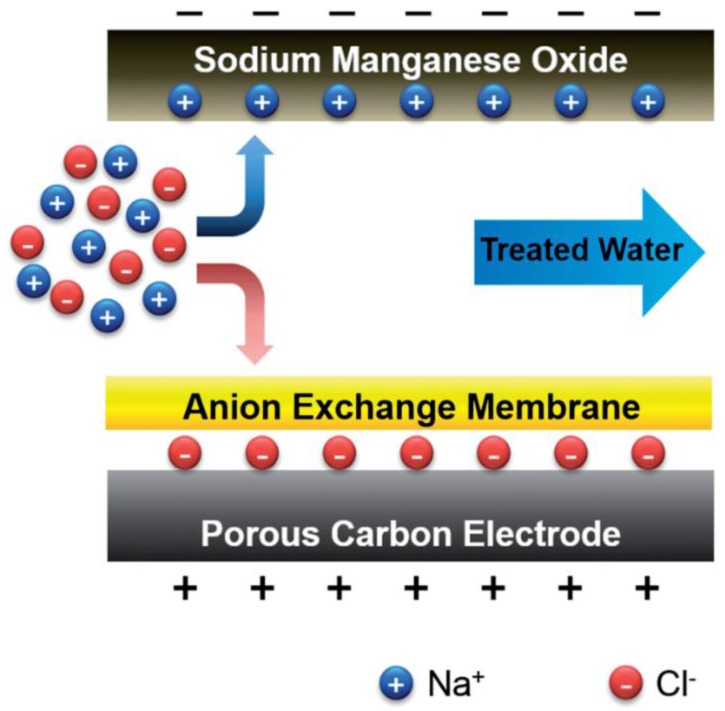
Schematic diagram of hybrid capacitive deionisation (HCDI). Reproduced with permission from Lee et al. [133], copyright Royal Society of Chemistry, 2014.

Lee et al. [133] replaced the cathode with a sodium manganese oxide electrode that benefited from higher specific capacity (Figure 11). In this approach, sodium ions are adsorbed into the sodium manganese electrode, while chloride ions adsorb in a standard format MCDI anode. 

Using this approach, Lee et al. [133] could improve the ion removal capacity from 13.5 mg g^−1^ in CDI and 22.4 mg g^−1^ of MCDI to 31.2 mg g^−1^ (per total mass of sodium manganese oxide and carbon). While the adsorption rate was higher than that of CDI, the performance was slower compared with regular MCDI. Further, the cost of these electrodes is likely to be well above those of simple activated carbon. As noted by Suss et al. [134], the ultimate performance-normalized costs associated with such novel CDI configurations requires assessment.

## 7. Conclusions and Future Research Directions

This article has provided an overview of the current status of MCDI, with a focus on the role of the ion exchange membrane in this process. Recent developments in the area of composite electrodes, novel IEMs, performance metrics, fouling and cleaning, and innovative configurations of MCDI were discussed in details. MCDI is a promising technique for desalination at low feedwater salinities, where the energy consumption appears lower than in comparable membrane processes, at least at the lab scale. However, further techno-economic studies comparing this approach to other desalination technologies including electrodialysis, nanofiltration, and reverse osmosis are of great importance to encourage more pilot-plant development and larger scale investments in the area. Additionally, while many research groups are enhancing the performance of MCDI by the fabrication of novel IEMs or composite electrode-IEM electrodes, or by introducing novel MCDI configurations, more attention must be paid to operational issues. Specifically, the performance of these materials must be evaluated with a much wider range of monovalent and multivalent salts, not just NaCl. Their stability must also be assessed in fouling systems where regular cleaning will be necessary. Such fouling is an important issue that can jeopardize the efficiency of MCDI at a commercial scale and is thus worthy of further investigation. Finally, while ion transport through IEMs was only briefly described in this paper, this research field requires in-depth analysis and mathematical modelling to better distinguish the role of the membrane in MCDI applications. 

## Figures and Tables

**Table 1:** Fabrication and characterization properties of different cation-exchange membranes (CEMs) and composite electrodes for use in membrane capacitive deionisation (MCDI) processes.

Materials	Polymer or Polymer Coating	Water Uptake (wt %)	IEC (meq g^−1^)	Electrical Resistance (Ω cm^2^)	Thickness of Polymer or Coating (μm)	Specific Capacitance (F g^−1^)	Ref.
**Commercial Membranes**	Fumasep FKS	12–15	0.8–1.2	2.0–4.5	120	-	Supplier
	Neosepta CMX	25–30	1.5–1.8	3.0	170	-	[9]
	Selemion CMV	25	2.4	-	120	-	[9]
	Dupont Nafion	16	0.9	1.5	117	-	[9]
**IEMs for MCDI**	NaSS-MAA-MMA	121	0.99	0.7	90–140	-	[20]
	PVDF-g-PSVBS	61	1.14	2	-	-	[21]
	Crosslinked Sulfonated polystyrene	30	0.9	0.37	25	-	[23]
**Composite Electrodes**	Polyethyleneimine (PEI)	-	-	-	-	52	[26]
	PVA/SSA-coated	-	-	0.67–1.17	-	97–116	[27]
	SG-CNFs ^a^	-	-	-	<1	117	[28]
	PVA/SSA-coated	-	-	0.64	10	0.74 F cm^−2^	[29]
	Sulfonated PPO ^b^	-	0.93	-	4.9	-	[30]
	Sulfonated BPPO ^c^ coated	-	-	-	-	-	[31]
	Sulfonated graphene	-	-	-	-	108	[32]
	PVA/SSA/SSA-MA	44	2.8	-	6.4	-	[33]

^a^ Sulfonated graphene-carbon nanofibres; ^b^ poly(phenlylene oxide); ^c^ Bromomethylated poly (2,6-dimethyl-1,4-phenylene oxide).

**Table 2:** Fabrication and characterization properties of different anion-exchange membranes (AEMs) and composite electrodes for MCDI process.

Materials	Polymer or Polymer Coating	Water Uptake (wt %)	IEC (meq g^−1^)	Electrical Resistance (Ω cm^2^)	Thickness of Polymer or Coating (μm)	Specific Capacitance (F g^−1^)	Ref.
**Commercial Membranes**	Fumasep FAS	15–3013–23	1.6–2.01.0–1.3	0.3–0.61.7–3.0	30135	-	Supplier
	Neosepta AMX	25–30	1.4–1.7	2.4	140	-	[9]
	Selemion AMV	19	1.9	2.8	120	-	[9]
**IEMs for MCDI**	Aminated PVDF-g-VBC	25	1.0	4.8	-	-	[34]
**Composite Electrodes**	Purolite	-	-	-	-	19	[24]
	dimethyldiallyl ammonium chloride	-	-	-	-	53	[26]
	Anion exchange resin	-	1.20	-	-	-	[35]
		-	2.9	-	~40	-	[36]
	Aminated PVA	-	1.76	-	20–40	180	[37]
	Aminated PVA	-	-	-	-	184	[38]
	Aminated PSF ^a^	-	1.0	5.2	-	-	[30]
	Aminated BPPO	-	-	-	-	-	[31]
	Aminated Graphene					91	[32]
	Aminated PSF	37	2.1	-	10.6	-	[33]

^a^ Polysulfone.

**Table 3:** A comparison of salt adsorption capacities from parallel studies of m-MCDI or MCDI versus Capacitive Deionisation (CDI).

Feed Salt Concentration	Adsorption Capacity (mg g^−1^)			
(mg L^−1^)	m-MCDI	MCDI	CDI	Reference
50	-	2.04	1.05	[41]
100	2.09	-	2.0	[31]
200	-	5.3	3.7	[40]
200	-	10.2	8.1	[29]
300	-	3.5	1	[43]
400	-	4.3	3	[44]
400	9.5	-	5.0	[28]
500	9.3	6.5	-	[26]
750	-	45.6	30.3	[45]
1000	9	6	1.9	[46]

## References

[1] Xu T. (2005). Ion exchange membranes: State of their development and perspective. J. Membr. Sci..

[2] Sata T. (2004). Ion Exchange Membranes: Preparation, Characterization, Modification and Application.

[3] Ran J., Wu L., He Y., Yang Z., Wang Y., Jiang C., Ge L., Bakangura E., Xu T. (2017). Ion exchange membranes: New developments and applications. J. Membr. Sci..

[4] Strathmann H. (2004). Ion-Exchange Membrane Separation Processes.

[5] Nagarale R.K., Gohil G.S., Shahi V.K. (2006). Recent developments on ion-exchange membranes and electro-membrane processes. Adv. Colloid Interface Sci..

[6] Merle G., Wessling M., Nijmeijer K. (2011). Anion exchange membranes for alkaline fuel cells: A review. J. Membr. Sci..

[7] Strathmann H. (1985). Electrodialysis and its application in the chemical process industry. Sep. Purif. Methods.

[8] Bazinet L. (2005). Electrodialytic phenomena and their applications in the dairy industry: A review. Crit. Rev. Food Sci. Nutr..

[9] Kentish S.E., Kloester E., Stevens G.W., Scholes C.A., Dumée L.F. (2015). Electrodialysis in aqueous-organic mixtures. Sep. Purif. Rev..

[10] Demirer O.N., Naylor R.M., Rios Perez C.A., Wilkes E., Hidrovo C. (2013). Energetic performance optimization of a capacitive deionization system operating with transient cycles and brackish water. Desalination.

[11] Li Y., Zhang C., Jiang Y., Wang T.-J., Wang H. (2016). Effects of the hydration ratio on the electrosorption selectivity of ions during capacitive deionization. Desalination.

[12] Porada S., Zhao R., van der Wal A., Presser V., Biesheuvel P.M. (2013). Review on the science and technology of water desalination by capacitive deionisation. Prog. Mater. Sci..

[13] Han L., Karthikeyan K.G., Anderson M.A., Wouters J.J., Gregory K.B. (2013). Mechanistic insights into the use of oxide nanoparticles coated asymmetric electrodes for capacitive deionisation. Electrochim. Acta.

[14] Lee J.-B., Park K.-K., Eum H.-M., Lee C.-W. (2006). Desalination of a thermal power plant wastewater by membrane capacitive deionization. Desalination.

[15] Biesheuvel P.M., Zhao R., Porada S., van der Wal A. (2011). Theory of membrane capacitive deionisation including the effect of the electrode pore space. J. Colloid Interface Sci..

[16] Zhao R., Porada S., Biesheuvel P.M., van der Wal A. (2013). Energy consumption in membrane capacitive deionisation for different water recoveries and flow rates, and comparison with reverse osmosis. Desalination.

[17] McRae W.A. (2000). Electroseparations, Electrodialysis. Kirk-Othmer Encyclopedia of Chemical Technology.

[18] He S., Liu L., Wang X., Zhang S., Guiver M.D., Li N. (2016). Azide-assisted self-crosslinking of highly ion conductive anion exchange membranes. J. Membr. Sci..

[19] Kariduraganavar M.Y., Nagarale R.K., Kittur A.A., Kulkarni S.S. (2006). Ion-exchange membranes: Preparative methods for electrodialysis and fuel cell applications. Desalination.

[20] Kwak N.-S., Koo J.S., Hwang T.S., Choi E.M. (2012). Synthesis and electrical properties of NaSS–MAA–MMA cation exchange membranes for membrane capacitive deionisation (MCDI). Desalination.

[21] Kang K.W., Hwang C.W., Hwang T.S. (2015). Synthesis and properties of sodium vinylbenzene sulfonate-grafted poly(vinylidene fluoride) cation exchange membranes for membrane capacitive deionisation process. Macromol. Res..

[22] Jeong K.S., Hwang W.C., Hwang T.S. (2015). Synthesis of an aminated poly(vinylidene fluride-g-4-vinyl benzyl chloride) anion exchange membrane for membrane capacitive deionisation(MCDI). J. Membr. Sci..

[23] Qiu Q., Cha J.-H., Choi Y.-W., Choi J.-H., Shin J., Lee Y.-S. (2017). Preparation of stable polyethylene membranes filled with crosslinked sulfonated polystyrene for membrane capacitive deionisation by *γ*-irradiation. Macromol. Res..

[24] Lee J.-B., Park K.-K., Yoon S.-W., Park P.-Y., Park K.-I., Lee C.-W. (2009). Desalination performance of a carbon-based composite electrode. Desalination.

[25] Hou C.-H., Liu N.-L., Hsu H.-L., Den W. (2014). Development of multi-walled carbon nanotube/poly(vinyl alcohol) composite as electrode for capacitive deionisation. Sep. Purif. Technol..

[26] Liu Y., Pan L., Xu X., Lu T., Sun Z., Chua D.H. (2014). Enhanced desalination efficiency in modified membrane capacitive deionisation by introducing ion-exchange polymers in carbon nanotubes electrodes. Electrochim. Acta.

[27] Kim J.-S., Choi J.-H. (2010). Fabrication and characterization of a carbon electrode coated with cation-exchange polymer for the membrane capacitive deionisation applications. J. Membr. Sci..

[28] Qian B., Wang G., Ling Z., Dong Q., Wu T., Zhang X., Qiu J. (2015). Sulfonated Graphene as Cation-Selective Coating: A New Strategy for High-Performance Membrane Capacitive Deionization. Adv. Mater. Interfaces.

[29] Kim Y.-J., Choi J.-H. (2010). Improvement of desalination efficiency in capacitive deionization using a carbon electrode coated with an ion-exchange polymer. Water Res..

[30] Kim J., Kim C., Shin H., Rhim J. (2015). Application of synthesized anion and cation exchange polymers to membrane capacitive deionization (MCDI). Macromol. Res..

[31] Lee J.-Y., Seo S.-J., Yun S.-H., Moon S.-H. (2011). Preparation of ion exchanger layered electrodes for advanced membrane capacitive deionization (MCDI). Water Res..

[32] Liu P., Wang H., Yan T., Zhang J., Shi L., Zhang D. (2016). Grafting sulfonic and amine functional groups on 3D graphene for improved capacitive deionization. J. Mater. Chem. A.

[33] Kim J.S., Jeon Y.S., Rhim J.W. (2016). Application of poly(vinyl alcohol) and polysulfone based ionic exchange polymers to membrane capacitive deionization for the removal of mono- and divalent salts. Sep. Purif. Technol..

[34] Jeong J.S., Kim H.S., Cho M.D., Kang H.R. (2016). Regeneration Methods of Capacitive Deionization Electrodes in Water Purification. U.S. Patent.

[35] Kim Y.-J., Choi J.-H. (2012). Selective removal of nitrate ion using a novel composite carbon electrode in capacitive deionization. Water Res..

[36] Yeo J.-H., Choi J.-H. (2013). Enhancement of nitrate removal from a solution of mixed nitrate, chloride and sulfate ions using a nitrate-selective carbon electrode. Desalination.

[37] Tian G., Liu L., Meng Q., Cao B. (2014). Preparation and characterization of cross-linked quaternised polyvinyl alcohol membrane/activated carbon composite electrode for membrane capacitive deionisation. Desalination.

[38] Gu X., Deng Y., Wang C. (2017). Fabrication of anion-exchange polymer layered graphene-melamine electrodes for membrane capacitive deionisation. ACS Sustain. Chem. Eng..

[39] Li H., Gao Y., Pan L., Zhang Y., Chen Y., Sun Z. (2008). Electrosorptive desalination by carbon nanotubes and nanofibres electrodes and ion-exchange membranes. Water Res..

[40] Kim Y.-J., Choi J.-H. (2010). Enhanced desalination efficiency in capacitive deionisation with an ion-selective membrane. Sep. Purif. Technol..

[41] Li H., Zou L. (2011). Ion-exchange membrane capacitive deionisation: A new strategy for brackish water desalination. Desalination.

[42] Liang P., Yuan L., Yang X., Zhou S., Huang X. (2013). Coupling ion-exchangers with inexpensive activated carbon fiber electrodes to enhance the performance of capacitive deionisation cells for domestic wastewater desalination. Water Res..

[43] Omosebi A., Gao X., Landon J., Liu K. (2014). Asymmetric electrode configuration for enhanced membrane capacitive deionisation. ACS Appl. Mater. Interfaces.

[44] Zhao Y., Wang Y., Wang R., Wu Y., Xu S., Wang J. (2013). Performance comparison and energy consumption analysis of capacitive deionization and membrane capacitive deionization processes. Desalination.

[45] Ding M., Shi W., Guo L., Leong Z.Y., Baji A., Yang H.Y. (2017). Bimetallic metal–organic framework derived porous carbon nanostructures for high performance membrane capacitive desalination. J. Mat. Chem. A.

[46] Duan F., Li Y., Cao H., Wang Y., Zhang Y., Crittenden J.C. (2015). Activated carbon electrodes: Electrochemical oxidation coupled with desalination for wastewater treatment. Chemosphere.

[47] Zhao R., Biesheuvel P.M., Miedema H., Bruning H., van der Wal A. (2010). Charge efficiency: A functional tool to probe the double-layer structure inside of porous electrodes and application in the modeling of capacitive deionisation. J. Phys. Chem. Lett..

[48] Porada S., Bryjak M., van der Wal A., Biesheuvel P.M. (2012). Effect of electrode thickness variation on operation of capacitive deionisation. Electrochim. Acta.

[49] Huyskens C., Helsen J., de Haan A.B. (2013). Capacitive deionisation for water treatment: Screening of key performance parameters and comparison of performance for different ions. Desalination.

[50] Choi J.-H. (2014). Comparison of constant voltage (CV) and constant current (CC) operation in the membrane capacitive deionisation process. Desalination Water Treat..

[51] Qu Y., Campbell P.G., Gu L., Knipe J.M., Dzenitis E., Santiago J.G., Stadermann M. (2016). Energy consumption analysis of constant voltage and constant current operations in capacitive deionisation. Desalination.

[52] Kim Y.-J., Hur J., Bae W., Choi J.-H. (2010). Desalination of brackish water containing oil compound by capacitive deionisation process. Desalination.

[53] Li H., Nie C., Pan L., Sun Z. (2012). The study of membrane capacitive deionisation from charge efficiency. Desalin. Water Treat..

[54] Wimalasiri Y., Mossad M., Zou L. (2015). Thermodynamics and kinetics of adsorption of ammonium ions by graphene laminate electrodes in capacitive deionisation. Desalination.

[55] Mossad M., Zou L. (2012). A study of the capacitive deionisation performance under various operational conditions. J. Hazard. Mater..

[56] Dlugolecki P., van der Wal A. (2013). Energy recovery in membrane capacitive deionisation. Environ. Sci. Technol..

[57] Kim Y.-J., Kim J.-H., Choi J.-H. (2013). Selective removal of nitrate ions by controlling the applied current in membrane capacitive deionisation (MCDI). J. Membr. Sci..

[58] Ryu T., Lee D.-H., Ryu J.C., Shin J., Chung K.-S., Kim Y.H. (2015). Lithium recovery system using electrostatic field assistance. Hydrometallurgy.

[59] Yoon H., Lee J., Kim S.-R., Kang J., Kim S., Kim C., Yoon J. (2016). Capacitive deionisation with Ca-alginate coated-carbon electrode for hardness control. Desalination.

[60] Choi J., Lee H., Hong S. (2016). Capacitive deionisation (CDI) integrated with monovalent cation selective membrane for producing divalent cation-rich solution. Desalination.

[61] Rice G., Barber A.R., O’Connor A.J., Stevens G.W., Kentish S.E. (2011). Rejection of dairy salts by a nanofiltration membrane. Sep. Purif. Technol..

[62] Garcia-Aleman J., Dickson J.M. (2004). Permeation of mixed-salt solutions with commercial and pore-filled nanofiltration membranes: Membrane charge inversion phenomena. J. Membr. Sci..

[63] Labbez C., Fievet P., Szymczyk A., Vidonne A., Foissy A., Pagetti J. (2003). Retention of mineral salts by a polyamide nanofiltration membrane. Sep. Purif. Technol..

[64] Schaep J., Vandecasteele C., Mohammad A.W., Bowen W.R. (1999). Analysis of the salt retention of nanofiltration membranes using the Donnan-steric partitioning pore model. Sep. Sci. Technol..

[65] Van Limpt B., van der Wal A. (2014). Water and chemical savings in cooling towers by using membrane capacitive deionisation. Desalination.

[66] Mikhaylin S., Bazinet L. (2016). Fouling on ion-exchange membranes: Classification, characterization and strategies of prevention and control. Adv. Colloid Interface Sci..

[67] Ong C.S., Goh P.S., Lau W.J., Misdan N., Ismail A.F. (2016). Nanomaterials for biofouling and scaling mitigation of thin film composite membrane: A review. Desalination.

[68] Piyadasa C., Ridgway H.F., Yeager T.R., Stewart M.B., Pelekani C., Gray S.R., Orbell J.D. (2017). The application of electromagnetic fields to the control of the scaling and biofouling of reverse osmosis membranes—A review. Desalination.

[69] Mossad M., Zou L. (2013). Study of fouling and scaling in capacitive deionisation by using dissolved organic and inorganic salts. J. Hazard. Mater..

[70] Zhang W., Mossad M., Zou L. (2013). A study of the long-term operation of capacitive deionisation in inland brackish water desalination. Desalination.

[71] Wang C., Song H., Zhang Q., Wang B., Li A. (2015). Parameter optimization based on capacitive deionisation for highly efficient desalination of domestic wastewater biotreated effluent and the fouled electrode regeneration. Desalination.

[72] Korngold E., De Korosy F., Rahav R., Taboch M.F. (1970). Fouling of anion-selective membranes in electrodialysis. Desalination.

[73] Lee H.-J., Moon S.-H., Tsai S.-P. (2002). Effects of pulsed electric fields on membrane fouling in electrodialysis of NaCl solution containing humate. Sep. Purif. Technol..

[74] Langevin M.-E., Bazinet L. (2011). Ion-exchange membrane fouling by peptides: A phenomenon governed by electrostatic interactions. J. Membr. Sci..

[75] Fidaleo M., Moresi M. (2006). Electrodialysis applications in the food industry. Adv. Food Nutr. Res..

[76] Lee H.-J., Hong M.-K., Han S.-D., Cho S.-H., Moon S.-H. (2009). Fouling of an anion exchange membrane in the electrodialysis desalination process in the presence of organic foulants. Desalination.

[77] Lindstrand V., Sundström G., Jönsson A.-S. (2000). Fouling of electrodialysis membranes by organic substances. Desalination.

[78] Chen G.Q., Eschbach F.I.I., Weeks M., Gras S.L., Kentish S.E. (2016). Removal of lactic acid from acid whey using electrodialysis. Sep. Purif. Technol..

[79] Diblíková L., Čurda L., Kinčl J. (2013). The effect of dry matter and salt addition on cheese whey demineralisation. Int. Dairy J..

[80] Sienkiewicz T., Riedel C.-L. (1986). Whey and Whey Utilization.

[81] Ayala-Bribiesca E., Pourcelly G., Bazinet L. (2006). Nature identification and morphology characterization of cation-exchange membrane fouling during conventional electrodialysis. J. Colloid Interface Sci..

[82] Ayala-Bribiesca E., Pourcelly G., Bazinet L. (2007). Nature identification and morphology characterization of anion-exchange membrane fouling during conventional electrodialysis. J. Colloid Interface Sci..

[83] Bazinet L., Montpetit D., Ippersiel D., Mahdavi B., Amiot J., Lamarche F. (2003). Neutralization of hydroxide generated during skim milk electroacidification and its effect on bipolar and cationic membrane integrity. J. Membr. Sci..

[84] Ayala-Bribiesca E., Araya-Farias M., Pourcelly G., Bazinet L. (2006). Effect of concentrate solution pH and mineral composition of a whey protein diluate solution on membrane fouling formation during conventional electrodialysis. J. Membr. Sci..

[85] Bleha M., Tishchenko G., Šumberová V., Kůdela V. (1992). Characteristic of the critical state of membranes in ED-desalination of milk whey. Desalination.

[86] Bazinet L., Araya-Farias M. (2005). Effect of calcium and carbonate concentrations on cationic membrane fouling during electrodialysis. J. Colloid Interface Sci..

[87] Casademont C., Farias M.A., Pourcelly G., Bazinet L. (2008). Impact of electrodialytic parameters on cation migration kinetics and fouling nature of ion-exchange membranes during treatment of solutions with different magnesium/calcium ratios. J. Membr. Sci..

[88] Trägårdh G. (1989). Membrane cleaning. Desalination.

[89] Katz W.E. (1979). The electrodialysis reversal (EDR) process. Desalination.

[90] Vermaas D.A., Kunteng D., Veerman J., Saakes M., Nijmeijer K. (2014). Periodic feedwater reversal and air sparging as antifouling strategies in reverse electrodialysis. Environ. Sci. Technol..

[91] Shee F.L.T., Angers P., Bazinet L. (2008). Microscopic approach for the identification of cationic membrane fouling during cheddar cheese whey electroacidification. J. Colloid Interface Sci..

[92] Sata T., Tsujimoto M., Yamaguchi T., Matsusaki K. (1996). Change of anion exchange membranes in an aqueous sodium hydroxide solution at high temperature. J. Membr. Sci..

[93] Komkova E.N., Stamatialis D.F., Strathmann H., Wessling M. (2004). Anion-exchange membranes containing diamines: Preparation and stability in alkaline solution. J. Membr. Sci..

[94] Vega J.A., Chartier C., Mustain W.E. (2010). Effect of hydroxide and carbonate alkaline media on anion exchange membranes. J. Power Sources.

[95] Garcia-Vasquez W., Ghalloussi R., Dammak L., Larchet C., Nikonenko V., Grande D. (2014). Structure and properties of heterogeneous and homogeneous ion-exchange membranes subjected to ageing in sodium hypochlorite. J. Membr. Sci..

[96] Ghalloussi R., Garcia-Vasquez W., Chaabane L., Dammak L., Larchet C., Deabate S.V., Nevakshenova E., Nikonenko V., Grande D. (2013). Ageing of ion-exchange membranes in electrodialysis: A structural and physicochemical investigation. J. Membr. Sci..

[97] Wang Q., Yang P., Cong W. (2011). Cation-exchange membrane fouling and cleaning in bipolar membrane electrodialysis of industrial glutamate production wastewater. Sep. Purif. Technol..

[98] Haddad M., Mikhaylin S., Bazinet L., Savadogo O., Paris J. (2016). Electrochemical acidification of kraft black liquor: Effect of fouling and chemical cleaning on ion exchange membrane integrity. ACS Sustain. Chem. Eng..

[99] Association A.W.W. (1995). Electrodialysis and Electrodialysis Reversal: M38.

[100] Guo H., You F., Yu S., Li L., Zhao D. (2015). Mechanisms of chemical cleaning of ion exchange membranes: A case study of plant-scale electrodialysis for oily wastewater treatment. J. Membr. Sci..

[101] Garcia-Vasquez W., Dammak L., Larchet C., Nikonenko V., Grande D. (2016). Effects of acid-base cleaning procedure on structure and properties of anion-exchange membranes used in electrodialysis. J. Membr. Sci..

[102] Biesheuvel P.M., Porada S., Levi M., Bazant M.Z. (2014). Attractive forces in microporous carbon electrodes for capacitive deionisation. J. Solid State Electrochem..

[103] Hassanvand A., Chen G.Q., Webley P.A., Kentish S.E. (2017). Improvement of MCDI operation and design through experiment and modelling: Regeneration with brine and optimum residence time. Desalination.

[104] Xie W., Cook J., Park H.B., Freeman B.D., Lee C.H., McGrath J.E. (2011). Fundamental salt and water transport properties in directly copolymerized disulfonated poly(arylene ether sulfone) random copolymers. Polymer.

[105] Geise G.M., Freeman B.D., Paul D.R. (2010). Characterization of a sulfonated pentablock copolymer for desalination applications. Polymer.

[106] Geise G.M., Paul D.R., Freeman B.D. (2014). Fundamental water and salt transport properties of polymeric materials. Prog. Polym. Sci..

[107] Sata T., Sata T., Yang W. (2002). Studies on cation-exchange membranes having permselectivity between cations in electrodialysis. J. Membr. Sci..

[108] Van der Bruggen B., Koninckx A., Vandecasteele C. (2004). Separation of monovalent and divalent ions from aqueous solution by electrodialysis and nanofiltration. Water Res..

[109] Galama A.H., Daubaras G., Burheim O.S., Rijnaarts H.H.M., Post J.W. (2014). Seawater electrodialysis with preferential removal of divalent ions. J. Membr. Sci..

[110] Ju H., Sagle A.C., Freeman B.D., Mardel J.I., Hill A.J. (2010). Characterization of sodium chloride and water transport in crosslinked poly(ethylene oxide) hydrogels. J. Membr. Sci..

[111] Crank J., Park G.S. (1968). Diffusion in Polymers.

[112] Pintauro P.N., Bennion D.N. (1984). Mass transport of electrolytes in membranes. 2. Determination of sodium chloride equilibrium and transport parameters for Nafion. Ind. Eng. Chem. Fundam..

[113] Helfferich F.G. (1995). Ion Exchange.

[114] Mackie J., Meares P. (1955). The diffusion of electrolytes in a cation-exchange resin membrane I. Theoretical. Proceedings of the Royal Society of London A: Mathematical, Physical and Engineering Sciences.

[115] Kamcev J., Paul D.R., Freeman B.D. (2015). Ion activity coefficients in ion exchange polymers: Applicability of Manning’s counterion condensation theory. Macromolecules.

[116] Kamcev J., Galizia M., Benedetti F.M., Jang E.-S., Paul D.R., Freeman B., Manning G.S. (2016). Partitioning of Mobile Ions Between Ion Exchange Polymers and Aqueous Salt Solutions: Importance of Counter-ion Condensation. Phys. Chem. Chem. Phys..

[117] Mackay D., Meares P. (1959). The electrical conductivity and electro-osmotic permeability of a cation-exchange resin. Trans. Faraday Soc..

[118] Kamo N., Toyoshima Y., Kobatake Y. (1971). Fixed charge density effective to membrane phenomena. Colloid Polym. Sci..

[119] Ueda T., Kamo N., Ishida N., Kobatake Y. (1972). Effective fixed charge density governing membrane phenomena. IV. Further study of activity coefficients and mobilities of small ions in charged membranes. J. Phys. Chem..

[120] Manning G.S. (1969). Limiting laws and counterion condensation in polyelectrolyte solutions II. Self-Diffusion of the small ions. J. Phys. Chem..

[121] Kamcev J., Paul D.R., Manning G.S., Freeman B.D. (2017). Predicting salt permeability coefficients in highly swollen, highly charged ion exchange membranes. ACS Appl. Mater. Interfaces.

[122] Curran P.M. (2015). Concentric Layer Electric Double Layer Capacitor Cylinder, System, and Method of Use. U.S. Patent.

[123] (2017). RDI™ Desalination System. http://www.atlantis-water.com/rdi-desalination-system-2/.

[124] Bian Y., Yang X., Liang P., Jiang Y., Zhang C., Huang X. (2015). Enhanced desalination performance of membrane capacitive deionisation cells by packing the flow chamber with granular activated carbon. Water Res..

[125] Jeon S.-I., Park H.-R., Yeo J.-G., Yang S.C., Cho C.H., Han M.H., Kim D.K. (2013). Desalination via a new membrane capacitive deionisation process utilizing flow-electrodes. Energy Environ. Sci..

[126] Gendel Y., Rommerskirchen A.K.E., David O., Wessling M. (2014). Batch mode and continuous desalination of water using flowing carbon deionisation (FCDI) technology. Electrochem. Commun..

[127] Porada S., Weingarth D., Hamelers H.V., Bryjak M., Presser V., Biesheuvel P. (2014). Carbon flow electrodes for continuous operation of capacitive deionisation and capacitive mixing energy generation. J. Mater. Chem. A.

[128] Yang S., Jeon S.-I., Kim H., Choi J., Yeo J.-G., Park H.-R., Kim D.K. (2016). Stack design and operation for scaling up the capacity of flow-electrode capacitive deionisation technology. ACS Sustain. Chem. Eng..

[129] Rommerskirchen A., Gendel Y., Wessling M. (2015). Single module flow-electrode capacitive deionisation for continuous water desalination. Electrochem. Commun..

[130] Yang S.-C., Choi J., Yeo J.-G., Jeon S.-I., Park H.-R., Kim D.K. (2016). Flow-electrode capacitive deionisation using an aqueous electrolyte with a high salt concentration. Environ. Sci. Technol..

[131] Ma J., He D., Tang W., Kovalsky P., He C., Zhang C., Waite T.D. (2016). Development of redox-active flow electrodes for high-performance capacitive deionisation. Environ. Sci. Technol..

[132] Hoyt N.C., Savinell R.F., Wainright J.S. (2016). Modeling of flowable slurry electrodes with combined Faradaic and nonfaradaic currents. Chem. Eng. Sci..

[133] Lee J., Kim S., Kim C., Yoon J. (2014). Hybrid capacitive deionisation to enhance the desalination performance of capacitive techniques. Energy Environ. Sci..

[134] Suss M., Porada S., Sun X., Biesheuvel P., Yoon J., Presser V. (2015). Water desalination via capacitive deionisation: What is it and what can we expect from it?. Energy Environ. Sci..

